# Molecular characterization of a microbial consortium involved in methane oxidation coupled to denitrification under micro-aerobic conditions

**DOI:** 10.1111/1751-7915.12097

**Published:** 2013-11-19

**Authors:** Jingjing Liu, Faqian Sun, Liang Wang, Xi Ju, Weixiang Wu, Yingxu Chen

**Affiliations:** 1 Institute of Environmental Science and Technology, Zhejiang UniversityYuhangtang Road 866#, Hangzhou, 310058, China; 2 Department of Architecture and Resources Engineering, Jiangxi University of Science and Technology Nanchang CompusShuanggang East Road 1180#, Nanchang, 310013, China; 3 Engineering Department, Jiangxi Zhongmei Engineering Construction Co., LtdNanlian Road 76#, Nanchang, 330001, China

## Abstract

Methane can be used as an alternative carbon source in biological denitrification because it is nontoxic, widely available and relatively inexpensive. A microbial consortium involved in methane oxidation coupled to denitrification (MOD) was enriched with nitrite and nitrate as electron acceptors under micro-aerobic conditions. The 16S rRNA gene combined with *pmoA* phylogeny of methanotrophs and *nirK* phylogeny of denitrifiers were analysed to reveal the dominant microbial populations and functional microorganisms. Real-time quantitative polymerase chain reaction results showed high numbers of methanotrophs and denitrifiers in the enriched consortium. The 16S rRNA gene clone library revealed that *Methylococcaceae* and *Methylophilaceae* were the dominant populations in the MOD ecosystem. Phylogenetic analyses of *pmoA* gene clone libraries indicated that all methanotrophs belonged to *Methylococcaceae*, a type I methanotroph employing the ribulose monophosphate pathway for methane oxidation. Methylotrophic denitrifiers of the *Methylophilaceae* that can utilize organic intermediates (i.e. formaldehyde, citrate and acetate) released from the methanotrophs played a vital role in aerobic denitrification. This study is the first report to confirm micro-aerobic denitrification and to make phylogenetic and functional assignments for some members of the microbial assemblages involved in MOD.

## Introduction

Denitrification is an important transformation in the cycling of nitrogen (N), which is ubiquitous and involves a stepwise reduction of nitrate (NO_3_^−^) to nitrogen oxides and nitrogen gas (N_2_). Biological removal of NO_3_^−^ and/or nitrite (NO_2_^−^) from wastewater, groundwater, landfill leachate and drinking water is commonly achieved by denitrification (Zumft, 1997). Supplemental organic carbon sources are generally added during denitrification, serving as electron donors, which adds cost to wastewater treatment processes. Methane (CH_4_) has been proposed as a relatively inexpensive, widely available, nontoxic carbon source, which is produced as a biogas by anaerobic waste processing and is available in many landfill sites and wastewater treatment plants. Denitrification using CH_4_ as an electron donor is an interesting option for biological nitrogen removal in this respect (Costa *et al*., 2000).

Methane oxidation coupled to denitrification (MOD) in the presence of oxygen (O_2_) has been shown in many studies. Eisentraeger and colleagues (2001) found that CH_4_ could be used as hydrogen donor for the in situ denitrification of groundwater. Waki and colleagues (2005) detected a denitrification rate of 4.29 mmol N l^−1^ day^−1^ using CH_4_ as the sole carbon source in a divided headspace reactor, where CH_4_ and air were sparged into opposite separate ends of an activated sludge tank (21% O_2_). Membrane biofilm reactors have been shown to achieve high NO_3_^−^ removal efficiencies compared with previously known rates for suspended cultures (Modin *et al*., 2008). It has been suggested that in the aerobic MOD process, methanotrophs oxidize CH_4_ aerobically and release organic compounds, which are then used by habiting denitrifiers as electron donors for anaerobic respiration (Modin *et al*., 2007). An association between methanotrophic and denitrifying bacteria surviving on CH_4_ as the sole carbon source and accomplishing denitrification was first demonstrated by Rhee and Fuhs (1978). They indicated that denitrifiers reduced NO_3_^−^ using citrate released from the CH_4_ oxidation. So far, previous studies have demonstrated directly or indirectly that methanotrophs can excrete methanol (Costa *et al*., 2000; Eisentraeger *et al*., 2001), citrate (Rhee and Fuhs, 1978), acetate (Costa *et al*., 2000), proteins (Eisentraeger *et al*., 2001), etc. to serve as the carbon and energy sources for denitrification. Although Osaka and colleagues (2008) revealed bacterial populations and methanotrophic populations by T-restriction fragment length polymorphism (RFLP) analysis of 16S rRNA genes and cloning of *pmoA* genes in an aerobic MOD ecosystem and many investigations of aerobic MOD have focused on denitrification, the mechanisms and microbial communities participating in these processes have not yet been confirmed.

Anaerobic denitrifying bacteria are characterized by the ability to use nitrogen oxides (NO_3_^−^ and NO_2_^−^) as electron acceptors producing dinitrogen monoxide (N_2_O) and dinitrogen (N_2_), which is inhibited by O_2_ (Knowles, 1982). However, some species are capable of simultaneously utilizing NO_3_^−^ and O_2_ as terminal electron acceptors (TEAs) in respiration, termed aerobic denitrification (Robertson and Kuenen, 1984). Although aerobic MOD has been investigated for several years, it is still unclear whether anaerobic or aerobic denitrification dominates in this process. Modin and colleagues (2007) put forward a theoretical model that indicated the optimal O_2_ dissolution rate in aerobic MOD systems should be as high as possible to maximize the methanotrophs' ability to oxidize CH_4_ and thereby provide organic substrates for coexisting denitrifiers but at the same time low enough for the dissolved oxygen (DO) concentration to remain at a level that does not inhibit denitrification. Thus, micro-aerobic conditions might be more favourable for the MOD process, especially for promoting denitrification activity. Nevertheless, to our knowledge, few or no studies have focused on the effect of both NO_2_^−^ and NO_3_^−^ in a micro-aerobic MOD system. Moreover, the microbial composition of the mixed cultures involved are not yet clarified.

The *pmoA* gene encoding the *α*-subunit of the particulate methane monooxygenase (pMMO) enzyme can be used as a functional phylogenetic marker to identify methanotrophic bacteria and is present in almost all known methanotrophs (Holmes *et al*., 1995). The reduction of NO_2_^−^ to nitric oxide (NO) by nitrite reductase is the first step that distinguishes denitrifiers from nitrate-respiring bacteria. Two structurally different nitrite reductases are found among denitrifiers: a copper-containing nitrite reductase encoded by the *nirK* gene and a cytochrome *cd*1-nitrite reductase encoded by the *nirS* gene (Zumft, 1997). The *pmoA* and *nirK* genes have been used as functional markers for cultivation-independent studies of the methanotrophic and denitrifying bacteria respectively (Lardy *et al*., 2010; Smith *et al*., 1987).

The objective of this study was to examine whether denitrification using NO_2_^−^ and NO_3_^−^ as electron acceptors with CH_4_ as the sole electron donor in a microaerophilic environment was possible and to indentify the associated intermediates, microbial community composition, and the primary microorganisms involved in this previously poorly characterized process. Experiments on the enrichment of methanotrophic bacteria under micro-aerobic conditions were performed using NO_2_^−^ and NO_3_^−^ as electron acceptors. Denitrification was assessed by quantifying of NO_2_^−^ and NO_3_^−^ consumption. In order to obtain insight into the microbial communities involved in MOD, a culture-independent DNA-based molecular phylogenetic approach was performed using the bacterial 16S rRNA genes, and *pmoA* and *nirK* gene clone libraries derived from the enrichment culture.

## Results

### Performance of the reactor and batch culture experiments

**Fig. 1:**
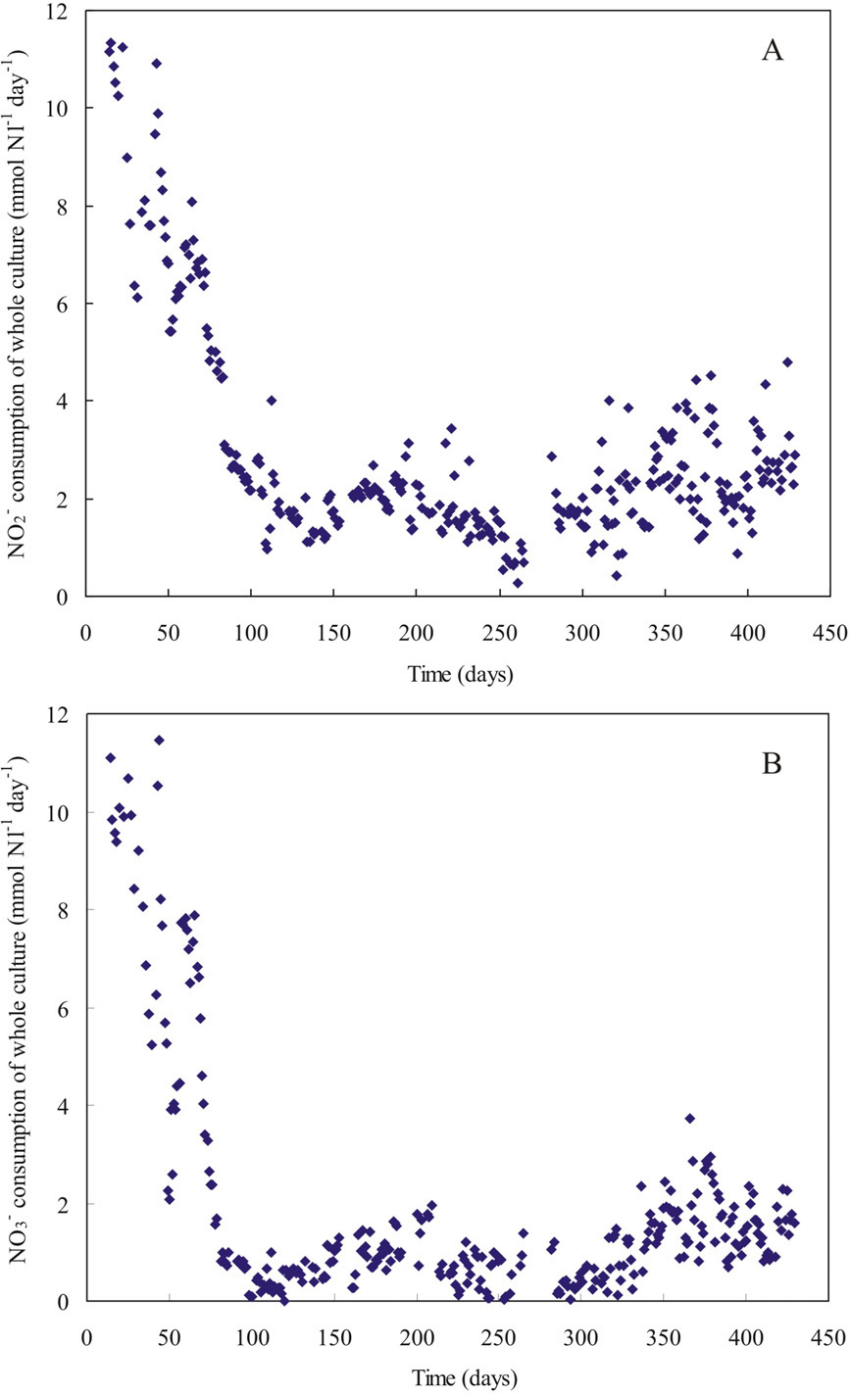
A. Nitrite consumption rates in the reactor with methane as the sole carbon source. B. Nitrate consumption rates in the bioreactor with methane as the sole carbon source.

The reactor was seeded with activated sediment from the 16 years old leachate collection pond of a landfill site. The reactor was operated for 430 days in the presence of CH_4_ as sole electron donor under micro-aerobic conditions. The effluent pH of the reactor was 7.2–7.5 after day 50. Because of the high concentration of ammonium (NH_4_^+^) in the seeded sediment, the effluent NH_4_^+^ decreased gradually to 0 after 100 days of incubation. Consumption of clear NO_2_^−^ and NO_3_^−^ was observed during the entire incubation period (Fig. 1). The consumption of NO_2_^−^ and NO_3_^−^ decreased gradually in the initial 100 days, while residual organic carbon sources were exhausted completely. From day 100 to 340, NO_2_^−^ and NO_3_^−^ consumptions increased slowly. In the steady phase, after incubation for 340 days, the effluent NO_2_^−^ and NO_3_^−^ concentrations remained stable at around 3.57 and 4.28 mM, with consumption rates approximately at 3.0 and 1.5 mmol N l^−1^ day^−1^ respectively.

The concentrations of CH_4_ and DO in the culture were around 0.23 and 0.22 mg l^−1^ respectively. There were obvious increases in soluble organic compounds between the influent and effluent liquid of the reactor. In particular, the concentrations of formaldehyde, citrate and acetate were greatly improved (Table 1).

**Table 1:** Organic carbon concentrations in the influent and effluent of liquid batch culture samples

Organic carbon	Influent (μg l^−1^)	Effluent (μg l^−1^)
Formaldehyde	11.50 ± 0.71	405.00 ± 7.07
Citrate	ND	236.67 ± 5.77
Acetate	22.50 ± 0.71	115.00 ± 7.07
Formate	0.95 ± 0.07	22.33 ± 1.53
Methanol	ND	0.40 ± 0.00

ND, not detected.

Methane oxidation activity and its coupling to denitrification were demonstrated in the batch experiments. The DO at T1 and T2 was 3.96 ± 0.09 and 0.24 ± 0.01 mg l^−1^ respectively. Volatile suspended solids in the sludge used in the assays were 24.98 ± 1.24 g l^−1^. Both of the treatments exhibited relatively high levels of CH_4_ oxidation and denitrification activity (Table 2) under both micro-aerobic and aerobic conditions. Furthermore, methane oxidation activity and denitrification activity under aerobic conditions (T1) were about twice higher than that measured under micro-aerobic conditions (T2). Compared with the control assay, both treatments resulted in high amounts of formaldehyde, citrate and acetate, which was in accordance with the results of the enrichment culture (Tables 1 and 2).

**Table 2:** Chemical activity in the batch culture experiments

Indexes	T1	T2	CK
CH_4_ : O_2_	90:10	99:1	–
Dissolved O_2_ (mg l^−1^)	3.96 ± 0.09	0.24 ± 0.01	–
Methane oxidation activity (μmol g VSS^−1^ day^−1^)	640.16 ± 88.76	354.21 ± 45.62	–
Denitrification activity (μmol g VSS^−1^ day^−1^)			
Nitrite consumption rate	68.39 ± 2.90	46.50 ± 6.19	28.03 ± 1.59
Nitrate consumption rate	25.50 ± 2.33	13.18 ± 2.07	5.02 ± 1.78
Formaldehyde (μg l^−1^)	355.00 ± 7.07	405.00 ± 7.07	19.50 ± 0.71
Citrate (μg l^−1^)	350.00 ± 14.14	320.00 ± 14.14	55.00 ± 7.07
Acetate (μg l^−1^)	130.00 ± 21.21	150.00 ± 0.00	17.2 ± 2.12
Formate (μg l^−1^)	24.70 ± 0.71	27.20 ± 1.41	0.6 ± 0.14
Methanol (μg l^−1^)	0.50 ± 0.00	0.55 ± 0.07	0.15 ± 0.07

VSS, volatile suspended solid.

### Phylogenetic analysis of bacterial communities based on the 16S rRNA gene clone library

**Fig. 2:**
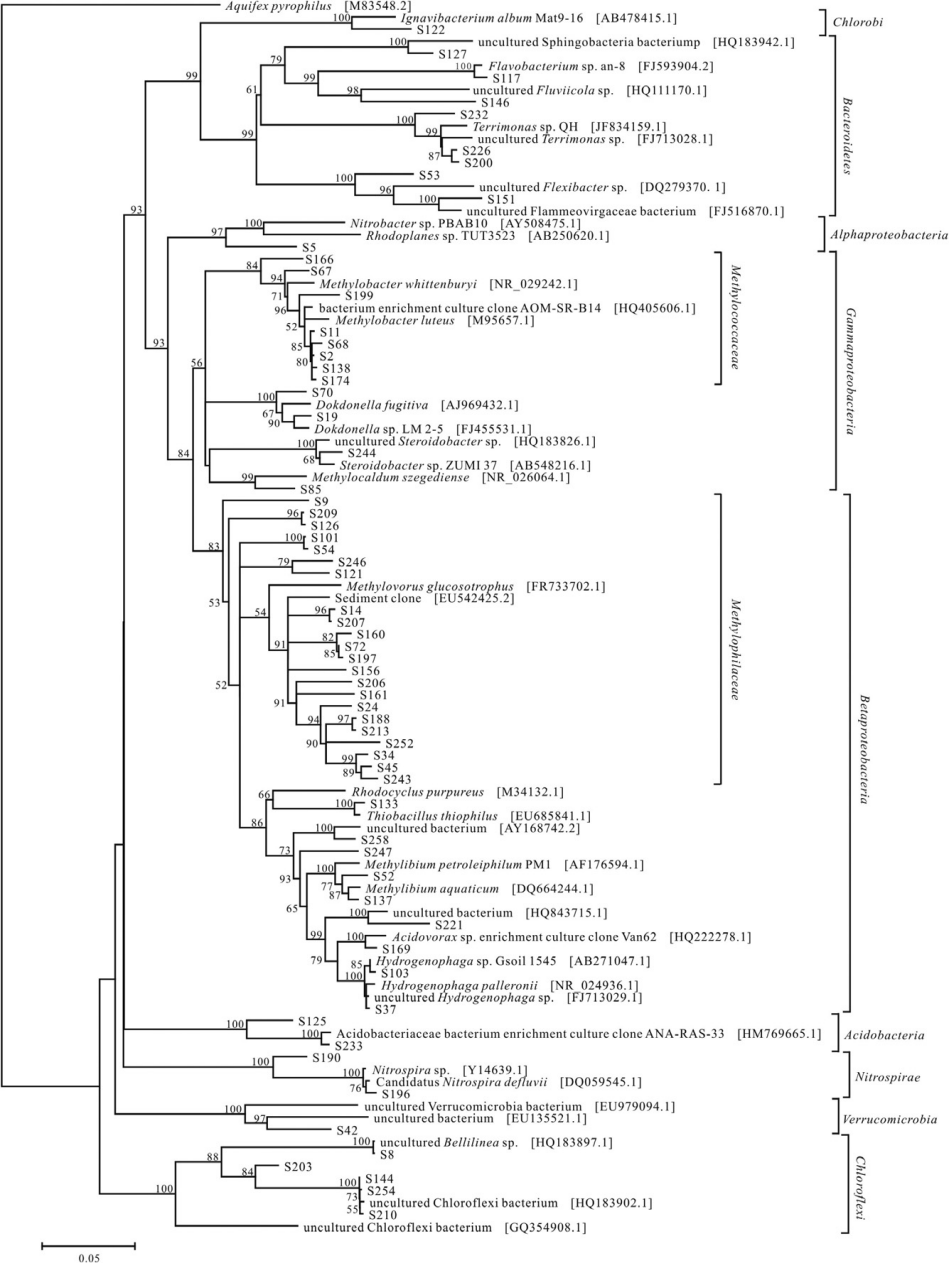
Phylogenetic relationships of phylotypes from bacterial 16S rRNA genes amplified from DNA extracted from enrichment culture samples. The 16S rRNA gene sequences were amplified with primer pair 27f and 1492r. The sequences were aligned with Clustal X; distance matrices and the phylogenetic tree were constructed using the Jukes-Cantor Model and neighbour-joining algorithms. Division level groupings are indicated on the right. *Aquifex pyrophilus* was used as the outgroup. GenBank accession numbers are in brackets. The numbers at the nodes are bootstrap confidence values expressed as percentages of 1000 bootstrap replications, and only values greater than 50% are reported. Bar = 0.05 change per sequence position.

A 16S rRNA gene clone library was constructed from total-community genomic DNA extracted from the enrichment culture after 1 year of cultivation, when the reactor was at a steady state. A total of 63 phylotypes affiliated with seven distinct phyla were examined among 136 randomly selected non-chimeric rDNA clones based on their RFLP patterns. The phylogenetic analysis of the phylotypes obtained was performed with reference sequences obtained from the GenBank database. The phylogenetic analyses showed that approximately 50% of bacterial sequences had close matches to counterparts in the public database (>98%). However, some clones displayed lower levels of similarity (<90%) to any other reported 16S rRNA gene sequences retrieved from GenBank database (Table 3). A distance-based neighbour-joining tree was constructed with the 63 phylotypes and their closely related reference sequences obtained from the GenBank database (Fig. 2).

**Table 3:** Phylogenetic affiliation of clones from the bacterial 16S rRNA gene library

Plylogenetic affiliation	No. of phylotypes	Proportion of phylotypes (%)	No. of clones	Proportion of clones (%)	% Sequence similarity to its closest relatives
*Bacteria*	63		136		
Proteobacteria	44	69.84	115	84.56	91.3–99.7
*α*-proteobacteria	1	1.59	1	0.74	94.2
*β*-proteobacteria	31	49.21	60	44.12	91.3–99.7
*γ*-proteobacteria	12	19.05	54	39.71	93.8–99.1
Bacteroidetes	8	12.7	10	7.35	87.8–99.8
Chloroflexi	5	7.94	5	3.68	91.8–99.8
Actinobacteria	2	3.17	2	1.47	91.6–99.2
Nitrospirae	2	3.17	2	1.47	91.7–98.9
Verrucomicrobia	1	1.59	1	0.74	88.0
Chlorobi	1	1.59	1	0.74	99.7

A majority of the clones belonged to the phylum *Proteobacteria* (115 of 136). The proteobacterial clones grouped into three classes, *Alphaproteobacteria*, *Betaproteobacteria* and *Gammaproteobacteria*. Most of the phylotypes were <98% identical to any previously determined rRNA gene sequences, although some of them were closely related to identified environmental clones. Overall, the proteobacterial phylotypes did not exhibit high phylogenetic diversity. The clone library showed a clear predominance of sequences related to *Methylophilaceae* in the *Betaproteobacteria* and to *Methylococcaceae* in the *Gammaproteobacteria*. Phylogenetic analyses indicated that the phylotypes associated with oxidation of CH_4_, oxidation of methanol and denitrification were all detected within the phylum *Proteobacteria*. The only phylotype belonging to *Alphaproteobacteria* was clone S5, remotely related (89.4% similarity) to *Nitrobacter* sp., which plays an important role in the nitrogen cycle by oxidizing NO_2_^−^ and NO_3_^−^.

Thirty-one phylotypes, representing 60 clones and accounting for 44% of the total number of clones, were related to the *Betaproteobacteria*. Twenty-two phylotypes, representing 50 clones, branched into a clade that was supported by strong bootstrap values within the family *Methylophilaceae*, although several of them were individually deep-branched in the phylogenetic tree. No closely related cultivated species were found for S72, the second most abundant *Betaproteobacteria*-related phylotype obtained. This phylotype, representing 16 clones, was associated phylogenetically (99.4% similarity) with a bacterial clone recently recovered from a sediment slurry (EU542425.2). Phylotypes S52 and S137 were closely affiliated with *Methylibium aquaticum*, a known chemoheterotrophic facultative methylotrophic bacterium isolated from an eutrophic freshwater pond previously (Song and Cho, 2007). Phylotype S9 was 92.6% identical to *Methylocaldum* sp. 05J-I-7 (EU275146.1), a methylotrophic bacterium retrieved previously from landfill upland soil, clustered with the *Betaproteobacteria*-related clones in a clade distinct from the *Gammaproteobacteria* on the tree, which was supported by strong bootstrap values. These results indicated that they were novel microorganisms enriched in the MOD enrichment culture. Phylotype S244 was most closely affiliated with the denitrifier *Steroidobacter* sp., an ecologically important bacterium with the ability to reduce NO_3_^−^ to N_2_O and further to N_2_ without any intermediate accumulation of NO_2_^−^ (Fahrbach *et al*., 2008). Phylotype S103 and S37 grouped within the genus *Hydrogenophaga*, which can metabolize NO_3_^−^ via heterotrophic denitrification (Willems *et al*., 1989).

The remaining 12 proteobacterial phylotypes, representing 55 clones and accounting for 40% of the total number of clones, were *Gammaproteobacteria* related. Of these, eight phylotypes representing 50 clones clustered within the *Methylobacter* genus and comprised 37% of the clone library. The most numerically dominant phylotype, S2, representing 39 clones and comprising 29% of the total number of clones, was 99.0% identical to an obligate methanotroph, *Methylobacter marinus*, which was isolated previously from seawater near a sewage outfall in California and utilizes methane and methanol as sole sources of carbon and energy (Lidstrom, 1988; Bowman *et al*., 1993). Phylotype S85 clustered with *Methylocaldum szegediense*, which can use methane as the sole source of carbon and energy (Bodrossy *et al*., 1997).

Moreover, the remaining 21 clones, represented by 19 phylotypes of nonmethanotrophs and nonmethylotrophs (i.e. *Bacteroidetes*, *Chloroflexi*, *Actinobacteria*, *Nitrospirae*, *Verrucomicrobia* and *Chlorobi*), were also detected in this library (15%) (Table 3). The phylotypes S190 and S196 clustered within the phylum *Nitrospirae* and branched into a clade with members of the genus *Nitrospira*, which are responsible for NO_2_^−^ oxidation in most wastewater treatment systems (Spieck *et al*., 2006).

### Phylogenetic analysis of methanotrophic populations based on the *pmoA* gene

**Fig. 3:**
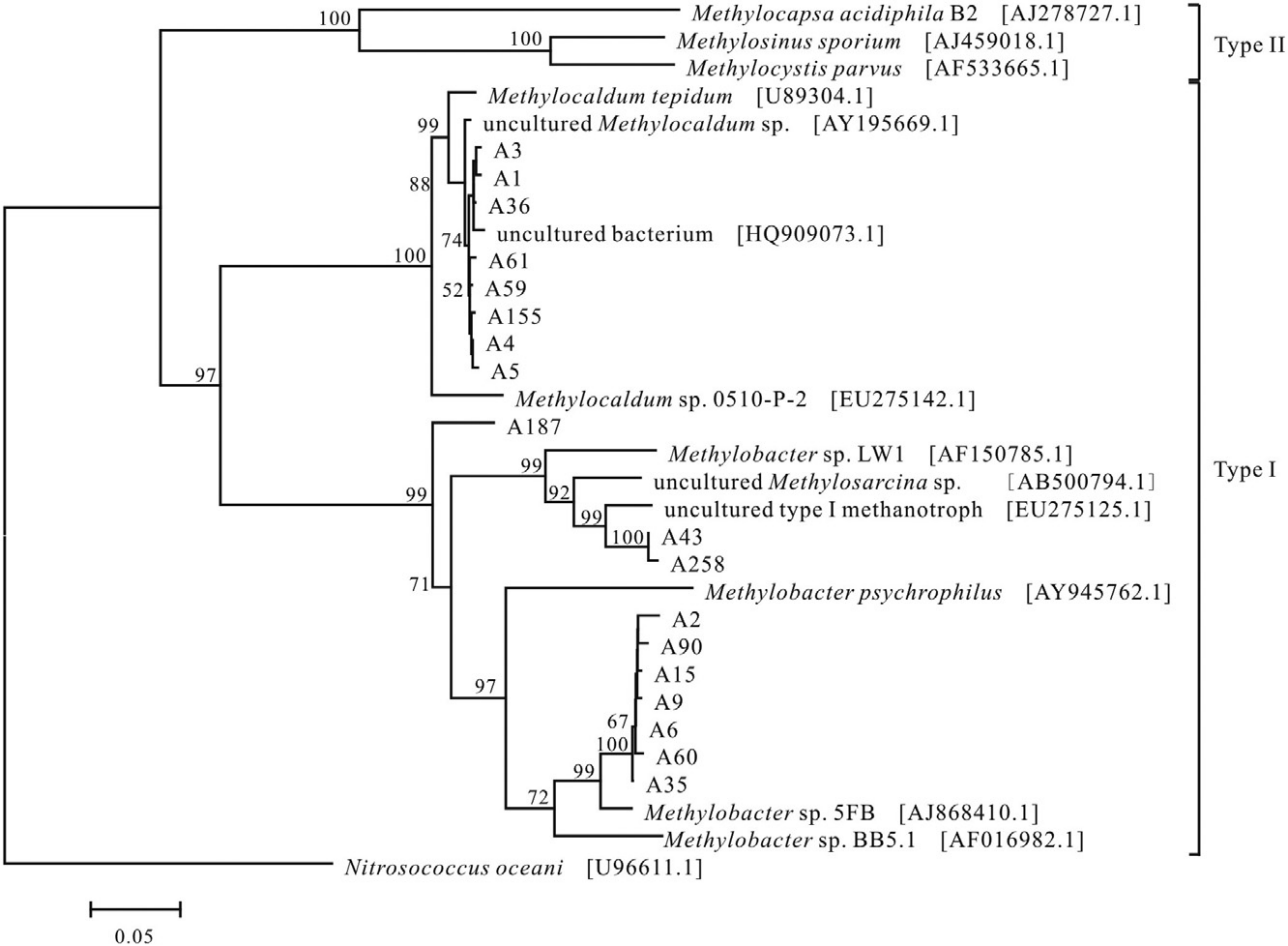
Phylogenetic relationships of *pmoA* genes amplified from DNA extracted from enrichment culture samples. The *pmoA* gene sequences were amplified with primer pair A189f and mb661r. The sequences were aligned with Clustal X; distance matrices and the phylogenetic tree were constructed by using the Jukes-Cantor Model and neighbour-joining algorithms. *Nitrosococcus oceani* was used as the outgroup. GenBank accession numbers are in brackets. The numbers at the nodes are bootstrap confidence values expressed as percentages of 1000 bootstrap replications, and only values greater than 50% are reported. Bar = 0.05 change per sequence position.

To characterize the methanotrophic population in the enrichment culture, the *pmoA* gene clone library was constructed from the same DNA templates as the 16S rRNA gene clone library. All the *pmoA* gene sequences clustered together on the phylogenetic tree, forming a monophyletic clade confirmed by bootstrap analysis (Fig. 3). The methanotrophic population as determined from the *pmoA* gene clone library was consistent with the results from the 16S rRNA gene clone library, although there were differences in the number of the *Methylocaldum*-related clones in these two clone libraries. A total of 18 phylotypes, examined among 229 *pmoA* gene clones, were affiliated with type I methanotrophs and fell into two groups within family *Methylococcaceae* of the *Gammaproteobacteria*. One group consisted of 109 clones, represented by 10 phylotypes, which belonged to the genus *Methylobacter*. However, all of the *Methylobacter* spp. exhibited relatively low level of similarity (<97%) with any sequences documented previously in the public databases. The other group included the remaining 120 clones, represented by eight phylotypes, which was closely related to the genus *Methylocaldum*, and they all displayed a high level of similarity (>99%) with their closest counterparts in public databases.

### Phylogenetic analysis of denitrifier populations based on the *nirK* gene

**Fig. 4:**
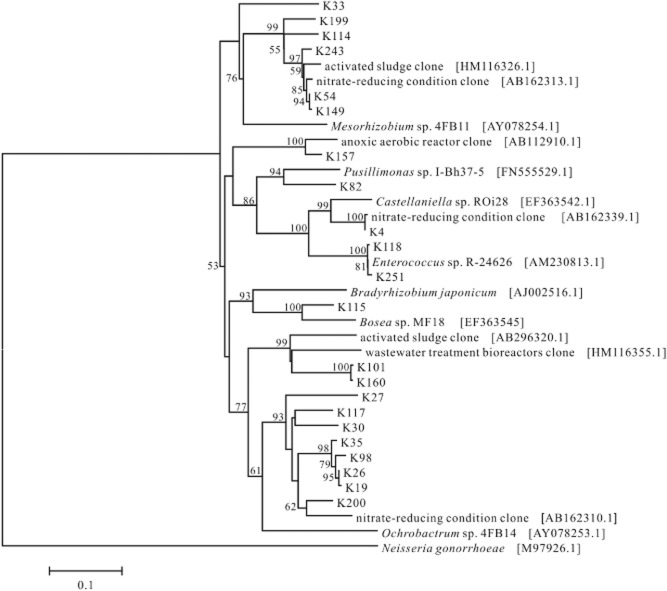
Phylogenetic relationships of *nirK* genes amplified from DNA extracted from enrichment samples. The *nirK* gene sequences were amplified with primer pair nirK1F and nirK5R. The sequences were aligned with ClustalX; distance matrices and phylogenetic trees were constructed by using the Jukes-Cantor Model and neighbour-joining algorithms. *Neisseria gonorrhoeae* was used as the outgroup. GenBank accession numbers are in brackets. The numbers at the nodes are bootstrap confidence values expressed as percentages of 1000 bootstrap replications, and only values greater than 50% are reported. Bar = 0.1 change per sequence position.

To characterize the denitrifying community in the enrichment culture, a *nirK* gene clone library was constructed from the same DNA templates used to construct the 16S rRNA gene clone library. A total of 22 phylotypes was examined from 52 randomly selected nonchimeric *nirK* gene clones as determined from RFLP patterns. The dendrogram of deduced amino acids showed three major clusters of *nirK* sequences (Fig. 4). Eighteen phylotypes representing 31 clones branched into two clades affiliated with *Alphaproteobacteria*. Most *Alphaproteobacteria*-related *nirK* clones were similar to uncultured bacteria or DNA sequenced from denaturing gradient gel electrophoresis bands obtained from a variety of environments, including activated sludge, biofilms, polluted water and soil. The remaining four phylotypes representing 21 *nirK* clones grouped within *Betaproteobacteria*. Of these, K251 and K118 were affiliated most closely (99.6% similarity) with *Enterococcus* sp. R24626, a denitrifier belonging to a less well-known denitrifying genus (Heylen *et al*., 2006). K4 was closely related (99.6% similarity) to the *nirK* sequence of an uncultured acetate-assimilating bacterium sampled under nitrate-reducing conditions (Osaka *et al*., 2006).

Although some of the *nirK* gene clones were closely related to cultured species (i.e. K251and K118), most of the *nirK* clones were distantly associated with the database sequences. BLAST (Basic Local Alignment Search Tool) comparisons showed that the homology between the *nirK* clones and database sequences ranged from 77.5–99.6%. Many phylotypes had remote phylogenetic association with other reported *nirK* sequences and were deeply branched in the phylogenetic tree, suggesting that these isolates might represent new species taxonomically.

### Quantification of methanotrophs and denitrifiers

Quantitative polymerase chain reaction (qPCR) was performed based on the V3 region of the 16S rRNA gene, *pmoA* gene and *nirK* gene sequences, to quantify the total bacteria, methane-oxidizing bacteria and denitrifying bacteria in the reactor after 1 year of enrichment cultivation. In the qPCR assay, the same DNA templates were used to construct the clone libraries. A high number (>10^9^) of gene copies were obtained for each of the three targeted genes, indicating that methanotrophs and denitrifiers were highly enriched in the reactor (Table 4).

**Table 4:** Quantification of the bacterial populations of the enrichment culture

Gene	No. of gene copies (× 10^9^ ml^−1^ sludge)
16S rRNA gene	13.71 ± 1.22
*pmoA* gene	8.71 ± 0.22
*nirK* gene	2.60 ± 0.07

## Discussion

MOD in the presence of O_2_ has been demonstrated in many studies (Thalasso *et al*., 1997; Eisentraeger *et al*., 2001; Modin *et al*., 2008). However, less is known about the mechanism and the key microbial populations responsible for this process. This study is the first to report that denitrification operated under micro-aerobic condition in which CH_4_ was used as the electron donor, and NO_2_^−^ and NO_3_^−^ as electron acceptors. Identifications of the chemical intermediates and the primary functional microorganisms are important steps in understanding the roles of different bacteria in this special process. In this study we characterized the phylogenetic composition of bacterial communities involved in MOD by 16S rRNA gene sequencing. Additionally, *pmoA* and *nirK* gene sequence analysis allowed us to identify some bacterial groups involved in these poorly studied but important biogeochemical processes.

A bacterial consortium performing MOD using both NO_2_^−^ and NO_3_^−^ as electron acceptors under micro-aerobic conditions was successfully enriched in this study. Steady NO_2_^−^ and NO_3_^−^ consumptions were observed in the enrichment culture, where only trace amounts of DO were detected in the culture medium. It has been demonstrated previously that some methanotrophs prefer micro-aerobic conditions (Mancinelli, 1995) and that methane-oxidizing enzymes have high affinities for O_2_ (Dalton, 1992). The batch experiment not only confirmed the occurrence of CH_4_ oxidation under micro-aerobic conditions but also indicated that more oxygen would likely promote methane oxidation activity. It has long been understood that DO generally suppresses denitrification activity because oxygen, when present, is the preferred TEAs for respiration. As a result, denitrifiers normally use NO_2_^−^ and NO_3_^−^ as TEAs only when oxygen is depleted in the environment (Zumft, 1997). However, we have shown that denitrification was not inhibited under our experimental conditions as the consumption of NO_2_^−^-N and NO_3_^−^-N was observed in both reactors in the batch experiments.

NO_2_^−^ and NO_3_^−^ consumption rates in T1 (90:10, CH_4_ : O_2_) were significantly higher than in T2 (99:1, CH_4_ : O_2_), which suggested that a higher concentration of O_2_ in the reactor could promote more efficient denitrification. It is possible that NO_2_^−^ and NO_3_^−^ depletion might be associated with aerobic denitrification because aerobic denitrifiers can co-respire by using NO_2_^−^ or NO_3_^−^, and O_2_ simultaneously as electron acceptors (Robertson *et al*., 1988). We did not measure N removal caused by assimilation, but no significant differences were observed in the concentrations of various organic compounds produced in T1 and T2, which suggests that denitrifiers exhibited higher activity and consumed more organic carbon in the aerobic treatment.

Methanotrophs in the enrichment culture could be classified into two genera within the family *Methylococcaceae* in the *Gammaproteobacteria* (i.e. *Methylobacter* and *Methylocaldum*) based on the phylogenetic analyses of *pmoA* and 16S rRNA genes sequences. The phylogenetic analyses showed that all of the methanotrophs in the enrichment culture belonged to type I methanotrophs, which employ the ribulose monophosphate (RuMP) pathway to assimilate formaldehyde (Wise *et al*., 1999). Type I methanotrophs in the family *Methylococcaceae* were the key functional populations in our MOD bioreactor. Previous studies have shown that a large number of methanotrophic bacteria that belong to the type II methanotrophs were present in an oxygen-limited MOD ecosystem (Costa *et al*., 2000) and that uncultured type X methanotrophs belonging to the *Gammaproteobacteria* were dominant in MOD incubations (Osaka *et al*., 2008). In general, type X methanotrophs grow at higher temperatures (Hanson and Hanson, 1996). The concentrations of CH_4_, O_2_ and inorganic nitrogen are typically the primary determinants of the type of methanotrophs present in an environment (Hanson and Hanson, 1996). Graham and colleagues (1993) demonstrated that the growth of type II methanotrophs was favoured under low-oxygen, high-methane conditions, and type I methanotrophs outcompeted type II methanotrophs at low-methane concentrations. Here, we enriched type I methanotrophs under low-oxygen, high-methane conditions. Type I methanotrophs tend to predominate under eutrophic conditions, whereas type II methanotrophs dominate in oligotrophic, nitrogen-limiting conditions because of its ability to fix nitrogen (Hanson and Wattenberg, 1991; Hanson and Hanson, 1996). Mohanty and colleagues (2006) demonstrated that nitrogenous fertilizers stimulated CH_4_ consumption and the growth of type I methanotrophs, whereas type II methanotrophs were inhibited. High levels of NO_2_^−^ and NO_3_^−^ in the bioreactor of this study stimulated the growth of type I methanotrophs. Different from type I methanotrophs, type II methanotrophs also assimilate carbon through the oxidation of formaldehyde but use the serine pathway (Wise *et al*., 1999). The RuMP pathway has been shown to be more efficient than the serine cycle, with conversion efficiencies of about 65–80% and 40–60% for methylotrophs using each of these pathways respectively (Anthony, 1982). Type I methanotrophs with their higher CH_4_ oxidation rate would have provided more soluble organic compounds for denitrifiers to reduce NO_2_^−^ and NO_3_^−^ to N_2_. Thus, we suggest that the abundance of nitrite and nitrate, and the presence of organic compounds for denitrifiers to respire were factors that allowed type I methanotrophs to outcompete type II methanotrophs in our MOD bioreactor.

At the same time, high numbers of nonmethanotrophic bacteria capable of denitrification were enriched along with the methanotrophic bacteria under our experimental conditions. From the enrichment culture and batch experiment, we infered that aerobic denitrification was conducted by certain species of bacteria that utilized O_2_, NO_2_^−^ and/or NO_3_^−^ simultaneously as electron acceptors. The existence and the growth of aerobic denitrifiers have been reported to occur in various environments (Frette *et al*., 1997; Huang and Tseng, 2001). In our batch experiments, enriched sludge in the aerobic treatment exhibited about twice as much denitrification activity compared with that in micro-aerobic treatment. It has been shown that aerobic denitrifiers grow more rapidly and denitrify more actively in the presence of both oxygen and NO_3_^−^ than in the presence of a single electron acceptor (Robertson *et al*., 1989). Furthermore, aerobic denitrifiers have been shown to use a variety of carbon sources as electron donors for oxygen respiration and denitrification, and that some of them prefer one-carbon compounds, such as methanol and formate (Takaya *et al*., 2003). Although there were no significant differences between the concentrations of organic compounds in micro-aerobic and aerobic treatments, we speculated that higher denitrification activity could consume more organic carbon, which reduced the amount of formaldehyde, citrate, acetate, etc. in the aerobic treatment. In the 16S rRNA gene clone library, the major phylotypes within nonmethanotrophs were related to the *Methylophilaceae*, which are strict facultative methylotrophs. Combined with phylogenetic analysis of the *nirK* gene clone library, we suggest that clones in our reactor belonging to the *Methylophilaceae* might be novel denitrifiers. Uncultured *Methylophilaceae* have been identified as important methylotrophic denitrifiers in sewage sludge (Osaka *et al*., 2006). Recently, some members of *Methylophilaceae* have been identified as aerobic methylotrophic denitrifiers in freshwater lake sediment, and they were demonstrated to be able to grow using one-carbon compounds, such as methanol, as carbon sources and nitrate as the TEAs under aerobic conditions (Kalyuhznaya *et al*., 2009). Our results indicated that these methylotrophic denitrifiers related to the *Methylophilaceae* facilitated denitrification in our MOD bioreactor.

Denitrifying bacteria can associate with methanotrophs and use simple carbon compounds released by the methanotrophs as substrates for denitrification reactions and growth in MOD. The soluble organic compounds may include methanol, citrate, acetate, proteins, etc. (Modin *et al*., 2007). In this study, analysis of the influent and effluent of the enrichment culture, as well as the batch experiments, showed that formaldehyde, citrate and acetate were the most prevalent compounds, which were released by the methanotrophic bacteria and supported growth and activity of denitrifiers. These organic compounds played a significant role as hydrogen donors for the indirect denitrification under CH_4_ oxidizing conditions. The compositions of the organic substances excreted by methanotrophs are highly dependent on the culture conditions and dominant methanotrophic species. How the methanotrophs regulate their carbon assimilation pathway might be confirmed by experiments using ^13^C-methane.

## Conclusions

The results presented in this paper confirm experimentally that aerobic denitrification can be performed with CH_4_ as the sole hydrogen donor under micro-aerobic conditions using NO_2_^−^ and NO_3_^−^ as electron acceptors. Composition of microbial communities and primary functional microorganisms in the MOD ecosystem bioreactor were investigated by a culture-independent DNA-based molecular phylogenetic approach. Clear NO_2_^−^ and NO_3_^−^ consumptions were observed using CH_4_ as sole electron donor during the culture process. Microbial community in the enrichment culture was dominated by *Methylophilaceae* and *Methylococcaceae*. Type I methanotrophs in the family of *Methylococcaceae* played a major role as key functional populations in MOD ecosystem under the micro-aerobic condition of this study. In addition to methanotrophic bacteria, large numbers of methylotrophic denitrifiers belonging to *Methylophilaceae* were enriched as well in the reactor implying their potential importance in aerobic denitrification. Formaldehyde, citrate and acetate were key substrates for trophic links between methanotrophs and denitrifiers in MOD ecosystems. The molecular phylogenetic analysis has given a first insight of the pivotal microbial populations and functional microorganisms involved in the micro-aerobic MOD ecosystem and can be used as a starting point for further studies.

## Experimental procedures

### Sampling and enrichment

Sediment samples were obtained in May 2009 from a 16 years old leachate collection pond at the Dongyang Landfill (29°14′49″N, 120°15′57″E), a waste disposal site for municipal solid waste in Zhejiang Province, PR China. The samples were transported to the lab and mixed with ambient leachate to obtain a homogeneous slurry (1 l) used for inoculation.

The sediment slurry was incubated in a 3 l plexiglass bioreactor, which was operated aseptically in a sequencing-batch mode to prevent loss of biomass. A 12 h cycle consisted of 10 h of stirring at 150 r.p.m., a settling period of 1.75 h and 0.25 h to draw off 150 ml of liquid from above the settled sediment. A 1.5 h continuous supply of 150 ml medium was included in the 12 h cycle. During the stirring time, the culture was stirred gently at 150 r.p.m., sparged with CH_4_, carbon dioxide (CO_2_) and O_2_ (94:5:1, v/v/v > 99.9%; flow rate, 10 ml min^−1^). The medium was sparged continuously with argon (Ar) and CO_2_ (95:5, v/v) to maintain anoxic conditions and contained the following components (l^−1^): 1.25 g KHCO_3_, 0.05 g KH_2_PO_4_, 0.30 g CaCl_2_·2H_2_O, 0.20 g MgSO_4_·7H_2_O, 0.425 g (5 mM) NaNO_3_, 0.345 g (5 mM) NaNO_2_, 1.0 ml acidic trace element solution, and 1.0 ml alkaline trace element solution. The acidic (100 mM HCl) trace element solution contained (l^−1^): 15 g ethylenediaminetetraacetic acid, 2.085 g FeSO_4_·7H_2_O, 0.068 g ZnSO_4_·7H_2_O, 0.12 g CoCl_2_·6H_2_O, 0.5 g MnCl_2_·4H_2_O, 0.32 g CuSO_4_, 0.095 g NiCl_2_·6H_2_O and 0.014 g H_3_BO_3_. The alkaline (10 mM NaOH) trace element solution contained (l^−1^): 0.067 g SeO_2_, 0.050 g Na_2_WO_4_·2H_2_O and 0.242 g Na_2_MoO_4_. The final concentrations of NO_2_^−^ and NO_3_^−^ in the medium were both 5 mM. The salts were dissolved in deionized water. All medium components were sterilized by autoclaving and mixed aseptically.

The minimum liquid volume of the enrichment culture was kept at 1.5 l by a level controller, and the maximum volume at the end of a filling period was 1.65 l. A gas buffer (l l) was filled with ultrapure water, which was used to prevent entry of air. The culture vessel was wrapped in black foil and run at 28 ± 1°C. The pH, NO_2_^−^ and NO_3_^−^ concentrations of the influent and effluent were analysed daily.

### Batch culture experiments

Batch culture experiments were carried out in 100 ml glass vials with butyl rubber stoppers and seals. The vials were filled with 10 ml medium sterilized by autoclaving and 8 ml enriched sludge. The medium contained the same components as the enrichment culture. Two treatments were performed. The gas headspace of the Treatment 1 (T1) vials was replaced with CH_4_-O_2_ (90:10, v/v > 99.9%) to maintain aerobic conditions; the gas headspace of the Treatment 2 (T2) vials were replaced with CH_4_-O_2_ (99:1, v/v > 99.9%) to create micro-aerobic conditions. Control assay was conducted in which the headspace of the vials was replaced with N_2_ (>99.99%). The CH_4_-dependent denitrification and the control assays were incubated in duplicate at 28 ± 1°C in the dark with shaking at 150 r.p.m. for 52 h. The concentration of CH_4_ in the headspaces, and NO_2_^−^, NO_3_^−^ and simple organic carbon concentrations in the batch cultures were measured at the beginning and the end of the incubations as described later.

### Analytical methods

All the chemical analyses were conducted according to standard methods (State Environmental Protection Administration of China, 2002). pH was measured by a pH analyser (Mettler Toledo, SG2, Greifensee, Switzerland). NO_3_^−^ concentration was determined by phenol disulfonic acid spectrophotometry, and NO_2_^−^ concentrations were determined with sulphanilamide and N-(1-naphthyl)-ethylenediamine spectrophotometrically. The concentrations of simple organic carbon molecules (i.e. formaldehyde, citrate, acetate, formate and methanol), O_2_ and CH_4_ were determined with a gas chromatography/mass spectrometry instrument (HP6890GC/5973MS, Agilent Technologies, Palo Alto, CA, USA). Every measurement was made in triplicate.

### Molecular analysis

#### DNA isolation

For the clone libraries, a 10 ml suspension was taken from the enrichment culture for DNA isolation after 1 year. Total DNA was extracted from approximately 0.5 ml of the sediment after allowing the suspension state to settle for 15 min using a beating method (FastDNA™ SPIN kit for soil, Bio101, Inc., La Jolla, CA, USA) following the manufacturer's protocol. DNA quality was checked by 1.5% agarose gel electrophoresis, and DNA extracts were stored at −20°C for downstream analyses.

#### PCR amplification

Bacteria 16S rRNA genes were amplified by PCR using the universal primer pair 27f-1492r (Lane, 1991). The PCR reaction mixture and amplification conditions were performed as described previously (Liu *et al*., 2011).

Primer pair A189-mb661 was used to amplify an internal region of the pMMO (Costello and Lidstrom, 1999), which specifically targets the *pmoA* gene. DNA was amplified in a Hybrid PCR Express thermal cycler (Bio-Rad, Hercules, CA, USA) in 0.2 ml PCR tubes using 50 μl reaction volumes. The reaction mixture contained the following components: 10 mM Tris-HCl, 50 mM KCl, 2.5 mM MgCl_2_, 0.2 mM of each deoxyribonucleoside triphosphate (dNTP), 0.4 μM of each primer (20 μM), 0.25 U of Taq DNA polymerase (TaKaRa, Dalian, China), 2 μl of template DNA. A touchdown PCR program was optimized and consisted of an initial denaturation step of 5 min at 94°C, followed by 16 touchdown cycles (60–52°C) and 18 further cycles at 52°C for 1 min, followed by 72°C for 1 min and a final extension of 72°C for 5 min.

Fragments of the nitrite reductase gene (*nirK*) were amplified using the specific primer pair nirK1F-nirK5R (Braker *et al*., 1998). PCR was run in a Hybrid PCR Express thermal cycler in 0.2 ml tubes using 20 μl reaction volumes. The reaction mixture contained the following components: 10 mM Tris-HCl, 50 mM KCl, 2.5 mM MgCl_2_, 0.2 mM of each dNTP, 0.4 μM of each primer (20 μM), 1 U of Taq DNA polymerase (TaKaRa), 2 μl of template DNA. DNA was denatured at 94°C for 5 min; 40 cycles of PCR were performed at 94°C for 30 s, 55°C for 30 s and 72°C for 40 s with a final extension at 72°C for 10 min.

The expected size of the fragments amplified was approximately 1460 bp for 16S rRNA gene, 470 bp for *pmoA* gene and 510 bp for *nirK* gene. Amplified products were analysed on a 1.5% agarose gel running in Tris-acetate-EDTA (TAE) buffer, stained with SYBR Green I (Invitrogen Biotechnology Co., Ltd, Shanghai, China) and ultraviolet illuminated.

#### Clone library construction and phylogenetic analysis

Amplified products were purified and subsequently cloned as described previously (Liu *et al*., 2011). Recombinant clones were randomly selected. Preliminary screening was done by directly reamplifying recombinant clones with the vector primer pair M13-47 and RV-M, and analysed for plasmids containing inserts by 1.5% agarose gel electrophoresis. The amplifications of positive clones were subjected to RFLP analysis by enzymatic digestions with restriction enzymes *Hha* I (Takara) following the manufacturer's instructions, and the digested nucleotide fragments were electrophoresed in 3% TAE-agarose gels. Clones were grouped according to RFLP banding patterns, and image analyses were performed visually to identify unique phylotypes. Sequencing was carried out by Invitrogen Corporation in China with an Applied Biosystems 3730xl DNA analyser.

All the sequences obtained in this work were checked for chimeras using the CHIMERA-CHECK online analysis program from Pintail (http://www.bioinformatics-toolkit.org). The chimeric sequences identified were not included in further phylogenetic analyses or clone library analyses. The nonchimeric sequences were submitted to the BLAST network service in the GenBank database (http://www.ncbi.nlm.nih.gov) to determine approximate phylogenetic affiliations (Altschul *et al*., 1997). Multiple alignments of the sequences from this study and reference sequences were performed using CLUSTAL X (Thompson *et al*., 1997). An average of at least 1480 nucleotides for the 16S rRNA gene, 470 nucleotides for the *pmoA* gene, and 510 nucleotides for the *nirK* gene was included in the phylogenetic analysis respectively. The phylogenetic trees were constructed based on a neighbour-joining algorithm (Saitou and Nei, 1987) in the MEGA4 computer software program (Tamura *et al*., 2007), using the Jukes-Cantor model (Jukes and Cantor, 1969). The confidence values of branches in the phylogenetic trees were determined using bootstrap analysis based on 1000 resamplings.

#### qPCR

Primer combinations of 338F-518R (Lane, 1991), A189-mb661 and nirK1F-nirK5R were used to quantify total bacteria, methane-oxidizing bacteria and denitrifying bacteria respectively. Real-time qPCR was performed with an iCycler iQ5 thermocycler and real-time detection system (Bio-Rad). For the standard curves, the plasmids were isolated and purified with the Plasmid Mini Kit (OMEGA Bio-Tek Inc., Doraville, GA, USA) from the clones in the relevant clone library. Each qPCR assay (25 μl) included 12.5 μl of 2 × SYBR Premix Ex Taq (Takara), 1 μl of each forward and reverse primer (20 μM), either 1 μl of template DNA or 10^−1^ to 10^−6^ ng of the standard vector plasmid of the clones grown as single cellular suspensions. The optimized thermal cycling was initiated with 3 min at 95°C, followed by 37 cycles of 15 s at 95°C, 30 s at 60°C (total bacteria and methane-oxidizing bacteria) or 55°C (denitrifying bacteria), and 30 s at 72°C. A melting curve analysis for the SYBR green assay was done after amplification to distinguish the targeted PCR products from the nontargeted PCR products.

### Nucleotide sequence accession numbers

The sequences of 16S rRNA genes have been deposited in the GenBank database under accession no. JN217030 to JN217092, *pmoA* gene sequences were assigned the accession numbers JN255553 to JN255570, and *nirK* gene sequences were assigned the accession numbers JN255526 to JN255547.
